# NUP98 – a novel predictor of response to anthracycline-based chemotherapy in triple negative breast cancer

**DOI:** 10.1186/s12885-019-5407-9

**Published:** 2019-04-02

**Authors:** Paul B. Mullan, Victoria Bingham, Paula Haddock, Gareth W. Irwin, Elaine Kay, Stephen McQuaid, Niamh E. Buckley

**Affiliations:** 1 Centre for Cancer Research and Cell Biology, Queen’s University Belfast, Belfast, BT9 7AE Northern Ireland; 2 School of Pharmacy, Queen’s University Belfast, Belfast, BT9 7BL Northern Ireland; 3 Nightingale Breast Centre, Wythenshawe Hospital, Manchester University Foundation Trust, Manchester, UK; 4 Department of Surgery, Beaumont Hospital and Royal College of Surgeons in Ireland, Dublin, Ireland

**Keywords:** Triple negative breast cancer, NUP98, Biomarker, Chemotherapy

## Abstract

**Background:**

Triple Negative breast cancer (TNBC) is a poor outcome subgroup of breast cancer defined based on the absence of expression of ERα and PR and HER2 amplification. These hard to treat cancers lack targeted treatment options and are therefore treated with a standard of care (SoC) generic cocktail of DNA damaging chemotherapy, with a wide range of clinical responses. While a subset of TNBC patients respond very well to this treatment, others receive no clinical benefit and die from their disease within a short time period. We currently lack biomarkers to prospectively identify patients likely to relapse and we lack alternate treatment options.

**Methods:**

NUP98 protein expression was investigated in patient samples using two independent tissue microarrays (TMAs), as well as a normal breast TMA. Correlation with pathological response to various chemotherapy regimens was investigated.

**Results:**

We have shown that high NUP98 is significantly associated with poor outcome in TNBC patient samples both by gene expression and IHC-based protein analysis. While trends linking NUP98 expression with poorer outcomes were observed in breast cancer overall (and more specifically in the LuminalB Her2- subgroup), significant correlations were observed in TNBC. This appeared to be specific to anthracycline based regimens as the association between NUP98 and response was not observed in patients treated with taxane-based chemotherapy.

**Conclusions:**

We have identified a novel biomarker, NUP98, that can predict response to anthracycline based chemotherapy in TNBC. The ability to prospectively identify patients who are less likely to respond to SoC chemotherapy is a vital step in improving the overall survival of these patients.

**Electronic supplementary material:**

The online version of this article (10.1186/s12885-019-5407-9) contains supplementary material, which is available to authorized users.

## Background

Breast cancer is a heterogeneous disease comprised of multiple tumour subtypes that require different treatment approaches and have varied patient outcomes. Patient stratification, based on the expression of the estrogen receptor (ERα) or amplification of the Her2/neu/ERBB2 (HER2) receptor, has facilitated the use of targeted therapies such as Tamoxifen and Trastuzumab, respectively. Breast cancers that do not express these receptors (as well as the Progesterone receptor (PR)) are termed “triple negative breast cancers” (TNBCs) and have the poorest clinical outcome, reflecting, in part, the fact that they lack targeted therapies. These hard to treat cancers are therefore treated with a SoC cocktail of DNA-damaging chemotherapies (e.g. FEC: 5-Fluorouracil, Epirubicin and Cyclophosphamide) with limited clinical response.

TNBCs display a unique clinical profile with very high risk of recurrence observed in the first 3 years following diagnosis, which then drops quickly to a rate lower than patient with non-TNBCs [1, 2]. While TNBC in general is associated with the poorest clinical outcome, neo-adjuvant studies have shown that there are at least 2 distinct subgroups of TNBC; one group displaying pathological complete response (pCR) following treatment and excellent survival rate comparable to non-TNBC cases with pCR. The second group, displaying residual disease (RD) following treatment, have much poorer outcome compared to non-TNBC with only 68% of patients alive 3 years post-treatment compared to 88% [2]. This demonstrates that overall survival in TNBC is intrinsically linked to the response to first line chemotherapy. However, we currently lack biomarkers to prospectively identify which patients are likely to respond to SoC and which are not and should have an alternate treatment plan.

Numerous studies have attempted to define further molecular subgroups within TNBC using unsupervised clustering of gene expression data in order to improve clinical outcome and identify novel treatment options and are summarised in [3]. In this study we instead used a supervised approach to identify genes associated with outcome in a cohort of TNBC all treated with SoC FEC chemotherapy [4]. We identified and validated the nuclear pore protein, NUP98, as a novel biomarker associated with poor outcome in TNBC in the context of SoC anthracycline based treatment. NUP98 has an established role in cancer as a fusion partner associated with leukaemogeneis. Conversely, it has been proposed as a tumour suppressor in liver cancer. Here we propose a novel oncogenic role for NUP98 in TNBC and highlight the potential utility not only as a biomarker in the context of SoC chemotherapy but also in novel targeted treatment strategies.

## Methods

Gene expression analysis: The QUB TNBC gene expression dataset has been previously described [4]. Public datasets, GSE6861, 7390, 9574, 22,093 and 20,271 were accessed online using NCBI. The ElasticNet regularization procedure was performed using the R package “glmnet” [5]. The optimal lambda (0.3) was chosen based on a 10-fold cross-validation. The ElasticNet regularization is a convex combination of the ridge and the lasso penalty with a weighting parameter “alpha”. Bootstrapping (× 100) followed by a hypergeometric test to identify non-random features was used for feature selection.

Survival Analysis: All Kaplan Meier analysis and Hazard Ratio Calculations were carried out using the R package “Survival”. Multivariate analysis was carried out using the clinical parameters: age, tumour grade, lymph node involvement, Lymphovascular invasion and chemotherapy regimen (in the case of the 2nd TNBC TMA).

### Tissue microarrays (TMAs) and immunohistochemistry (IHC)

The breast cancer TMAs used in this study were constructed from formalin-fixed paraffin-embedded (FFPE) primary tumour blocks by the Northern Ireland Biobank with each tumour sample represented by three independent 1 mm diameter cores. The full breast cohort [6] and the 2nd TNBC TMA [7] have been previously described with additional information provided in Additional file 1 Table S1.

The normal breast TMA consisted of 40 FFPE samples collected from reduction mammoplasty and/or normal adjacent tissue to cancer within Beaumont Hospital, Dublin. Whole face sections of the FFPE samples were stained with haematoxylin and eosin (H&E) to select epithelial rich regions for targeted coring. All cases were reviewed by an experienced breast histopathologist. The TMA was constructed using a manual tissue arrayer (Beecher Instruments, Silver Spring, MD, USA) with 1-mm-diameter tissue cores from donor blocks inserted into recipient blocks with each patient sample represented by three independent cores.

IHC was performed in a hybrid laboratory (Northern Ireland Molecular Pathology Laboratory) that has UK Clinical Pathology Accreditation, and the infrastructure to process both clinical patient samples and research materials. Sections were cut from the TMA blocks for H&E staining and IHC. The initial section was used for H&E staining to assess TMA quality and appropriate tumour content for subsequent IHC localization and analysis. Sections for IHC were cut at 4 μm on a rotary microtome, dried at 37 °C overnight, and then used for IHC, performed on an automated immunostainer (Leica Bond-Max, Milton Keynes, UK). Repeat ERα, PR and HER2 IHC were performed to confirm the triple negative status all samples in the TMA as previously described [6]. The 2 NUP antibodies were validated in-house using positive and negative whole-face breast cancer sections identified through gene expression. Antigen-binding sites were detected with a polymer-based detection system (Bond, Newcastle Upon Tyne, UK, Cat. No. DS 9800). All sections were visualized with diaminobenzidine, counterstained with haematoxylin, and mounted in DPX. Biomarker conditions were as follows. NUP98 - rabbit polyclonal antibody, LSBIO #B10323 was used at a 1:200 dilution with epitope retrieval solution 1 pre-treatment for 20 mins. NUP96 - rabbit monoclonal (clone EPR6678) antibody LSBIO #C138875 was used at a 1:50 dilution with epitope retrieval solution 2 for 20 mins.

### Scoring and assessment

Only cores with identifiable tumour as confirmed by pathology assessment of H&E slides were used in IHC analysis. All IHC was scored independently by at least two experienced immunohistochemists (SMcQ, NB, VB) blinded to patient clinicopathological and outcome data. Expression of both biomarkers was exclusively confined to the tumour epithelial cells. Individual TMA cores were scored as absent/low (0), intermediate [1], or high [2] expression of NUP98 based on intensity of staining.

Statistical Analysis: Statistical Analysis was carried out using GraphPad Prism (v6.0). All relevant data was analysed by two-tailed Students t-test or one-way Anova as required. Quantification of the relationship between NUP98 and pathological outcome was performed using contingency tables and analysis by Fisher’s exact test or chi-square test as appropriate. All data was deemed significant with a *p*-value of at least < 0.05 with * indicating a p-value < 0.05, ** a *p*-value of < 0.01 and *** a *p*-value of < 0.001.

## Results

**Fig. 1:**
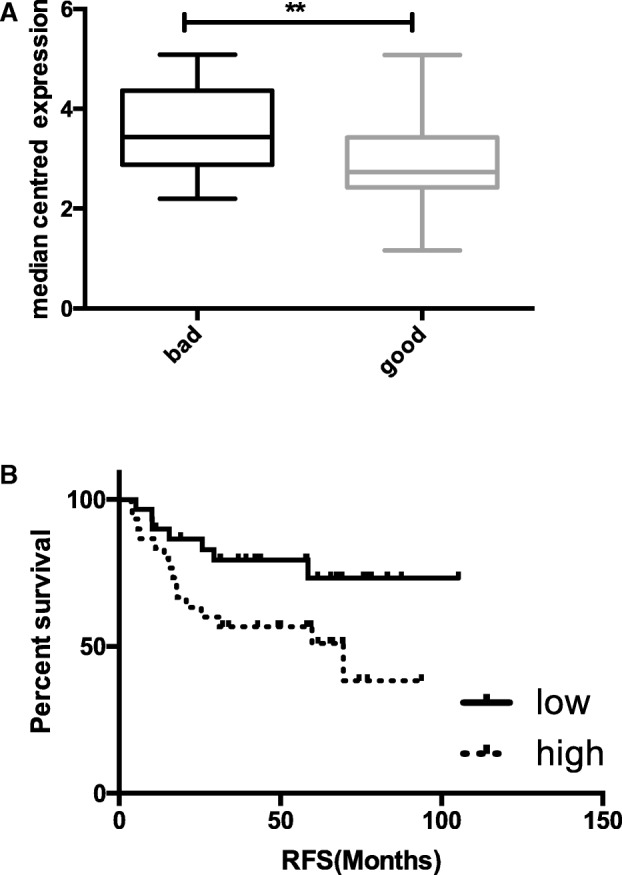
**a** Box and whisker plot of NUP98 gene expression in good and poor outcome samples in the in-house TNBC gene expression dataset. **b** Kaplan Meier plot of relapse free survival dichotomised based on NUP98 gene expression above (high) and below the median (low)

In order to identify genes associated with good (no relapse within 3 years post treatment) and poor (relapse within 3 years post treatment) outcome in TNBC, we used an elasticnet-based approach to identify genes whose expression was differentially associated with outcome in a cohort of 60 TNBC cases all treated with SoC chemotherapy (FEC). NUP98 was identified with significantly higher expression in the poor outcome samples (Figure 1a). Furthermore, high NUP98 expression (above the median) was also shown to be significantly associated with worse relapse free survival (RFS) using both univariate (HR 2.87 (95% CI 1.118–7.447) *p* = 0.0285) and multivariate (HR 4.885 (95% CI 1.6520–14.453) *p* = 0.00414) analysis, independent of all clinical variables (Fig. 1a and Tables 1 and 2). A similar trend was observed for overall survival (OS) though this did not reach significance (Additional file 2 Figure S1). However, given the fact that there is no cure for secondary breast cancer and time to death following relapse is shorter in TNBC compared to other types of breast cancer [1], it can be assumed that given a longer follow-up period, the findings for OS would mirror those seen for RFS.

**Table 1:** Cox Proportional Hazard ratios, 95% Confidence intervals (95%CI) and *p*-values for survival analysis of relapse free and overall survival dichotomised based on below (low) and above (high) the median gene expression of NUP98

Relapse Free Survival		N(n)	HR	%95 CI	*p*-value
Univariate		60 (21)			
Nup98	low	30 (6)	1		
	high	30 (15)	2.8856	1.118–7.447	0.0285*
Multivariate					
Nup98	low	30 (6)	1		
	high	30 (15)	4.885	1.6520–14.453	0.00414 **
Overall Survival		60 (16)			
Nup98	low	30 (6)	1		
	high	30 (10)	1.808	0.6738–4.791	0.2430

**Table 2:** Clinical characteristics of the NUP98 high and low samples within the in-house TNBC gene expression datasets

		Nup98 low (*N* = 30)	Nup98 high (*N* = 30)
Age	< 40	3	5
	40–49	11	12
	50–59	6	6
	60+	10	7
T code	1	11	9
	2–4	19	21
N code	0	18	21
	1–3	12	9
LVI	present	14	15
	Not seen	16	15

**Fig. 2:**
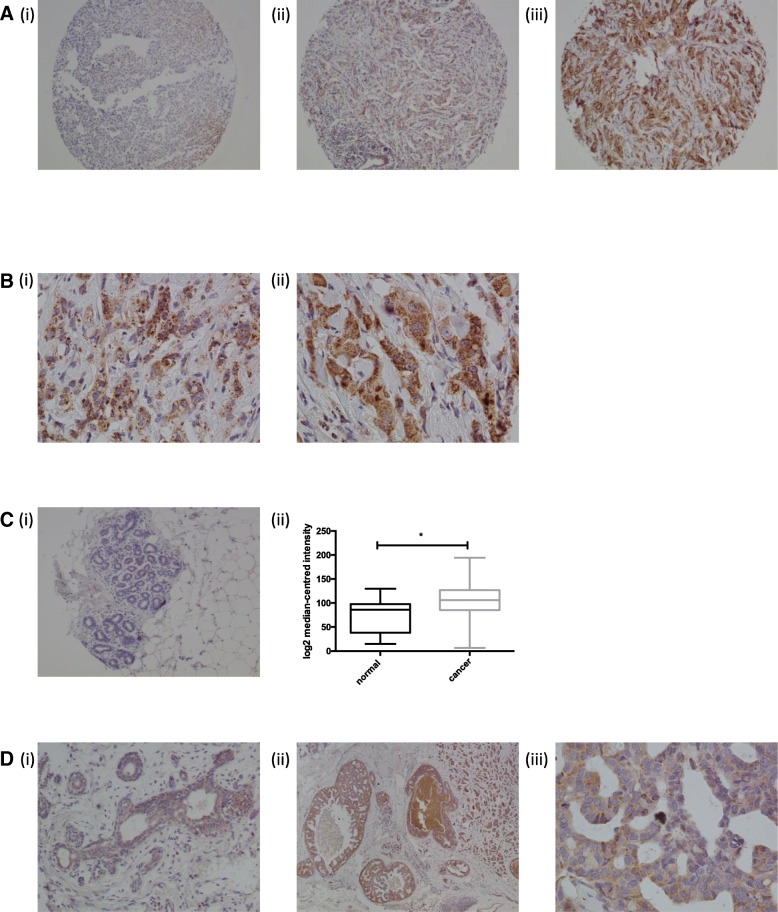
**a** Representative images demonstrating scoring strategy with (i) absent (score = 0), (ii) low (score = 1) and (iii) high (score = 2) NUP98 IHC expression. **b** Representative images of NUP98 staining at (i) × 20 and (ii) × 40 magnification. **c** (i) Representative image of absence of staining for NUP98 in normal breast tissue. (ii) Box and whisker plot of NUP98 expression in normal and cancer breast samples from publicly available dataset GSE9574. **d** Representative images at (i&ii) × 10 and (iii) × 40 magnification of NUP98 expression in DCIS

In order to further investigate the potential role for NUP98 as a biomarker of response to SoC, we next optimised and validated a commercially available antibody for use on tissue microarrays (TMAs) to assess NUP98 protein expression by immunohistochemistry (IHC). We first utilised a TMA with matched samples to the gene expression study. A range of expression of NUP98 was observed with low/absent (score = 0), intermediate (score = 1) and high (score = 2) expression patterns observed (Figure 2a). When staining was present, a punctate cytoplasmic pattern was observed within the epithelial compartment with most of the cells staining to a similar extent (Figure 2b). We also investigated NUP98 expression in a small TMA of normal breast tissue (*N* = 40) with no expression of NUP98 observed other than a rare cell with weakly positive staining in the nucleus (Figure 2c (i)). This was consistent with the statistically higher NUP98 expression observed in cancer vs normal samples from a publicly available dataset [8] (Figure 2c (ii)). Of interest, a few examples of DCIS were available for analysis within cores on the breast cancer TMA. NUP98 was expressed on the DCIS component of these cases (Figure 2d), suggesting that this is an early event in cancer.

**Fig. 3:**
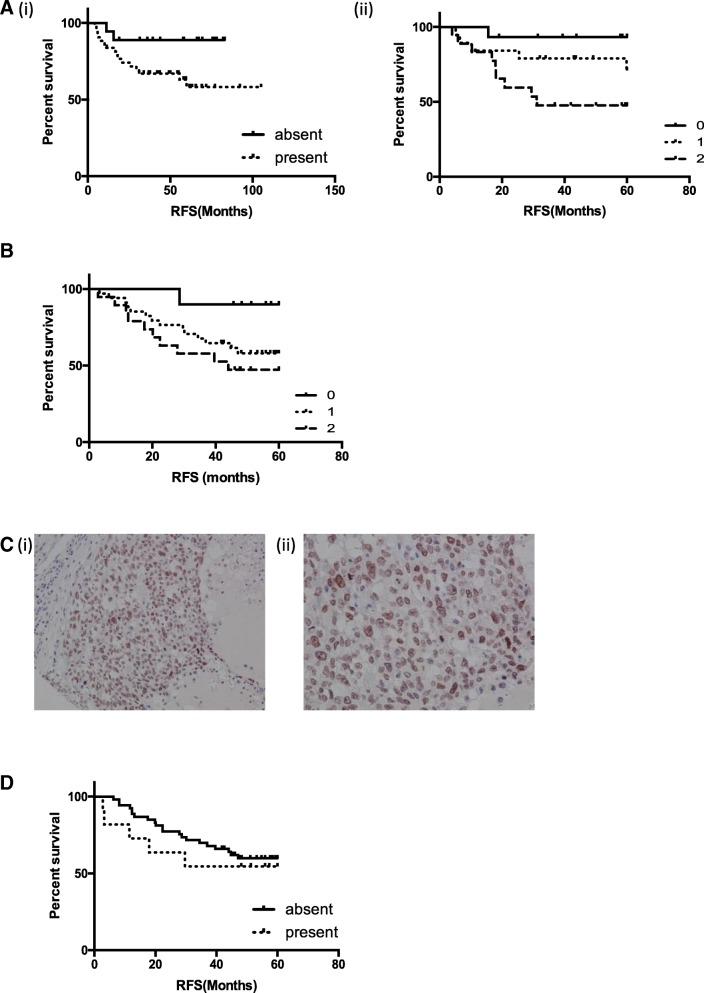
**a** Kaplan Meier plot of relapse free survival stratified based on (i) presence or absence, (ii) or absent (0), low (1) or high (2) NUP98 IHC expression in the in-house TNBC TMA1 with matched gene expression. **b** Kaplan Meier plot of relapse free survival stratified based on absent (0), low (1) or high (2) NUP98 IHC expression in the in-house TNBC TMA2. **c** Representative images of NUP96 staining at (i) × 10 and (ii) × 40 magnification. **d** Kaplan Meier plot of relapse free survival stratified based on presence or absence of NUP96

Consistent with the gene expression analysis, any (intermediate or high) NUP98 protein expression was associated with an almost significant worse relapse free survival (HR 6.707 (95%CI 0.8815–51.04) *p* = 0.066) (Figure 3a (i), Table 3a). Interestingly, this appeared to be a graduated effect with the highest NUP98 expression most significantly associated with poor outcome (HR 10.373 (95%CI 1.3035–82.54) *p* = 0.0271) (Figure 3a (ii), Table 3b). As seen with the gene expression data, a trend towards overall survival was also observed (Additiona file 2 Figure S1B, Table 2b).

**Table 3:** (A) Cox Proportional Hazard ratios, 95% Confidence intervals (95%CI) and p-values for survival analysis of relapse free survival dichotomised based on presence or absence and of NUP98 IHC expression in the in-house TNBC TMA1 with matched gene expression. (B) Cox Proportional Hazard ratios, 95% Confidence intervals (95%CI) and *p*-values for univariate and multivariate survival analysis of relapse free and overall survival stratified based on absent (0), low (1) or high (2) NUP98 IHC expression in the in-house TNBC TMA1 with matched gene expression. Cox Proportional Hazard ratios, 95% Confidence intervals (95%CI) and *p*-values for univariate and multivariate survival analysis of (C) relapse free and (D) overall survival stratified based on absent (0), low (1) or high (2) NUP98 IHC expression in the in-house TNBC TMA2. (E) Cox Proportional Hazard ratios, 95% Confidence intervals (95%CI) and p-values for survival analysis of relapse free survival dichotomised based on presence or absence and of NUP96 IHC expression in the in-house TNBC TMA2

**A**					
TNBC TMA #1		N(n)	HR	%95 CI	*p*-value
Relapse Free Survival		53(16)	Univariate		
Nup98	absent	15 (1)	1		
	present	37 (14)	6.707	0.8815–51.04	0.066
**B**					
TNBC TMA #1		N(n)	HR	%95 CI	*p*-value
Relapse Free Survival		53(16)	Univariate		
Nup98	low	15 (1)	1		
	medium	19 (5)	4.24	0.495–36.33	0.1871
	high	18 (9)	10.373	1.3035–82.54	0.0271*
Relapse Free Survival			Multivariate		
Nup98	low	15 (1)	1		
	medium	19 (5)	3.7522	0.41815–33.670	0.2375
	high	18 (9)	13.32	1.39448–127.242	0.0245*
Overall Survival		53 (12)	Univariate		
Nup98	low	15 (1)	1		
	medium	19 (3)	2.5638	0.266–24.65	0.415
	high	18 (6)	5.8375	0.7016–47.57	0.103
**C**					
TNBC TMA #2		N(n)	HR	%95 CI	p-value
Relapse Free Survival		63(24)	Univariate		
Nup98	low	10 (1)	1		
	medium	34 (14)	5.044	0.6629–38.38	0.1181
	high	19 (9)	7.293	0.9324–57.04	0.0583
			Multivariate		
Nup98	low	10 (1)	1		
	medium	34 (14)	4.39	0.051–37.87	0.1783
	high	19 (9)	8.95	1.06–75.11	0.0433*
**D**					
TNBC TMA #2		N(n)	HR	%95 CI	p-value
Relapse Free Survival		63(24)	Univariate		
Nup98	low	10 (1)	1		
	medium	34 (14)	5.086	0.6685–38.70	0.1162
	high	19 (9)	6.558	0.8293–51.86	0.0747
			Multivariate		
Nup98	low	10 (1)	1		
	medium	34 (14)	4.71	0.552–40.11	0.1566
	high	19 (9)	7.91	0.94–66.62	0.0570
**E**					
TNBC TMA #2		N(n) 53(16)	HR	%95 CI	p-value
Relapse Free Survival					
Nup96	absent	53(21)	1		
	present	11(5)	1.378	0.4839–4.234	0.5173
Overall Survival					
Nup96	absent	53 (20)	1		
	present	11 (5)	1.418	0.4943–4.445	0.04825*

Given the significant association between NUP98 and survival, we went on to validate our findings in a second independent TNBC cohort [7] as outlined in the REMARK guidelines [9]. A similar expression pattern was observed with low, intermediate and high expression (data not shown). Furthermore, a similar association between NUP98 expression and survival was observed with the highest expression associated with the worst survival which trended towards significance in a univariate analysis but did reach significance when analysed in a multivariate fashion (HR 8.95 (95%CI 1.06–75.11) *p* = 0.0433)(Figure 3b, Table 3c). Similar, though non-significant results were also seen when analysed for overall survival (Additional file 2 Figure S1C, Table 2d).

Four isoforms of NUP98 are present in human cells, all generated by alternate splicing of the gene [10]. Isoforms 1 and 4 are generated through splicing at exon 20 followed an unusual biogenesis pathway in which a large precursor protein is produced. This is then proteolytically cleaved to produce both NUP98 and NUP96 [11]. Isoforms 2 and 3 are generated without splicing at exon 20 and do not encode NUP96. As it was not possible to distinguish between isoforms through gene expression analysis or IHC, we also assessed NUP96 expression in our TNBC TMA to determine whether NUP96 was also associated with outcome. NUP96 was expressed in 19% of cases with a nuclear pattern of expression within the epithelial compartment with little variety in staining intensity (Figure 3c). Therefore, samples were designated as present or absent for NUP96 expression. There was no significant association between NUP96 expression and survival (Figure 3d, Additional file 2 Figure S1D, Table 3e).

**Fig. 4:**
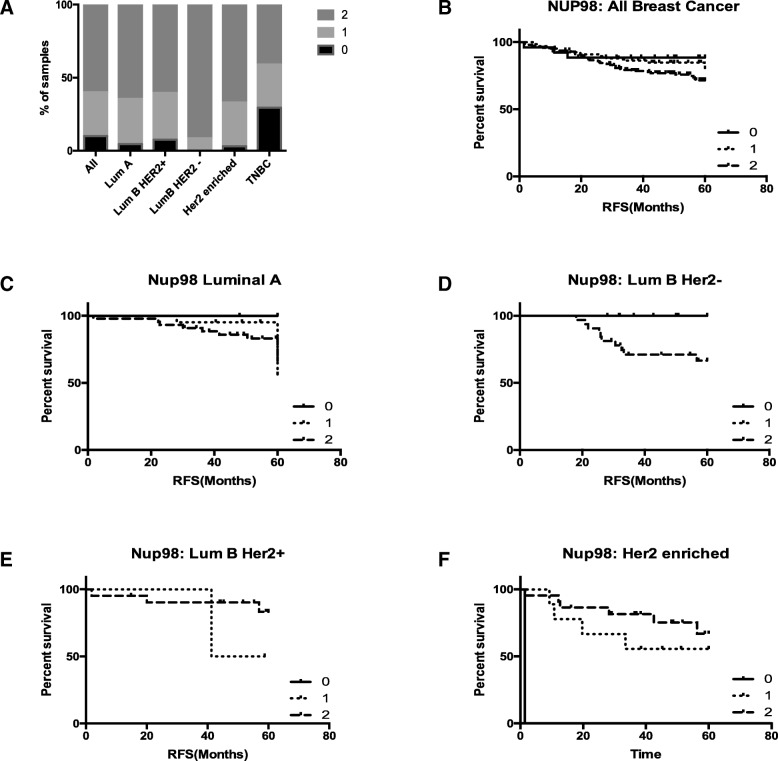
**a** Percentage distribution of NUP98 IHC expression in breast cancer overall, as well as subtypes defined by the St Gallen Classification method in the full Breast (*N* = 300) cohort. **b**-**f** Kaplan Meier plots of relapse free survival stratified based on absent (0), low (1) or high (2) NUP98 IHC expression in the St Gallen subgroups with the full Breast cohort

We next wanted to investigate whether the role for NUP98 as a biomarker to predict response to SoC was restricted to TNBC or could be applied to breast cancer as a whole. We therefore utilised the full cohort (*N* = 300) from which our original TNBC cases had been selected which represents breast cancer as a whole [6]. NUP98 was expressed (regardless of intensity) in the vast majority of cases (90%) with a punctate cytoplasmic pattern observed consistent with our previous TMA cohorts. However, the distribution of NUP98 expression varied significantly (chi-squared p = < 0.0001) between subtypes of breast cancer (defined by the St Gallen surrogate classification method [12]) with only the TNBC samples showing a substantial proportion of samples with no NUP98 expression (Figure 4A). NUP98 protein expression was then analysed in the context of survival in breast cancer as a whole as well as within the subtypes (Figures 4b-f). In general, high NUP98 expression was associated with worse outcome. Similar to our findings in TNBC (Figure 3a (ii)), high NUP98 expression was also associated with worse outcome in the Luminal B,HER2 negative subgroup. However, the graduation effect observed with intermediate expression was not seen (Figure 4d). Interestingly, in the HER2 enriched subgroup, high NUP98 expression was associated with a more favourable outcome indicating a unique role in this subgroup (Figure 4f). Due to the varying results in the different molecular subgroups, we focussed our subsequent investigations on TNBC as this demonstrated the most significant findings.

**Fig. 5:**
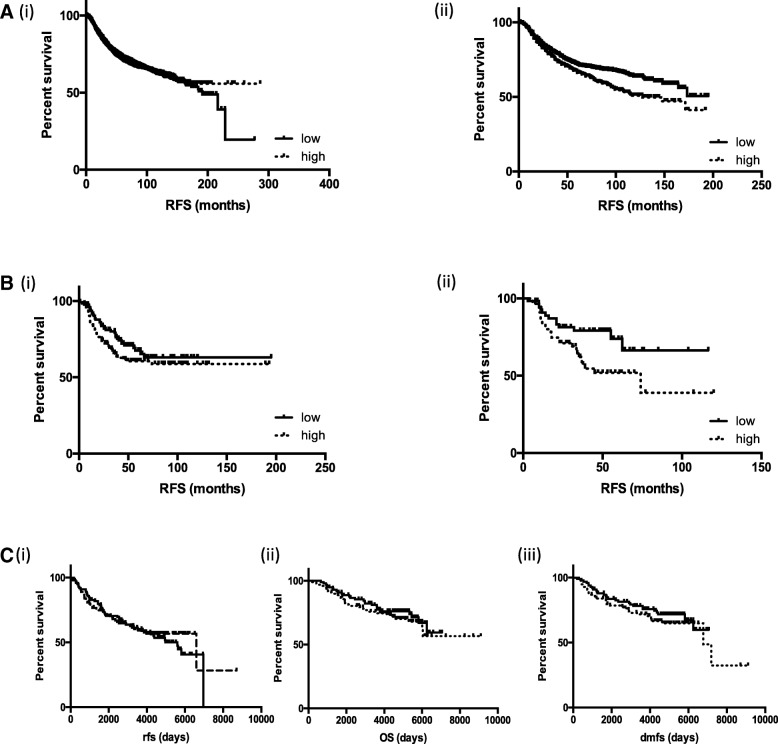
Kaplan Meier plot of relapse free survival dichotomised based on NUP98 expression above (high) and below the median (low) in (**a**)(i) all breast cancer samples, (**a**)(ii) all breast cancer samples excluding patients without systemic chemotherapy treatment, (**b**)(i) all TNBC samples and (**b**)(ii) all TNBC samples excluding patients without systemic chemotherapy treatment using KMplotter. **c** Kaplan Meier plot of (i) relapse free, (ii) overall survival and (iii) distant metastasis free survival in the systemically untreated early breast cancer TRANSBIG cohort (GSE7390)

Considering that all patients within our discovery (and most in the validation) cohort were treated with chemotherapy, we wanted to ascertain whether the association with NUP98 and survival was purely prognostic or predictive of response to treatment with SoC chemotherapy. Using the online tool, KM Plotter [13], a significant association with poor relapse free survival and high NUP98 expression was only observed when patients who did not receive systemic treatment were excluded in both breast cancer as a whole (Figure 5a) and TNBC (Figure 5b, Table 4). This was confirmed using analysis the TRANSBIG study, a dataset of early breast cancer where patients received no cytotoxic chemotherapy [14], where no association between NUP98 expression and RFS, DMFS or OS was observed (Figure 5c (i)-(iii)). These results indicated that NUP98 expression was predictive of outcome only in the context of chemotherapy. In order to understand if this was specific to the type of chemotherapy given, we analysed a dataset where patients were neo-adjuvantly treated with either a non-taxane- (FEC) or taxane-based (TET) regimen [15]. A highly significant association between NUP98 expression and pathological complete response (pCR) (*p* = 0.0057) was observed in patients treated with FEC – patients with low NUP98 expression were over twice as likely to achieve pCR as patients with high NUP98. No association between NUP98 expression and response to TET was observed (Table 5 and Additional file 1 Table S2). Similar results were seen in other neo-adjuvant cohorts treated with FEC or FAC [16, 17] (Table 5 and Additional file 1 Table S1) indicating a role for NUP98 in predicting response to non-taxane, anthracycline based chemotherapy.

**Table 4:** Cox Proportional Hazard ratios, 95% Confidence intervals (95%CI) and p-values for survival analysis of relapse free survival dichotomised based on NUP98 expression above (high) and below the median (low) in (A)(i) all breast cancer samples, (A)(ii) all breast cancer samples excluding patients without systemic chemotherapy treatment, (B)(i) all TNBC samples, (A)(ii) all TNBC samples excluding patients without systemic chemotherapy treatment

KMplotter Relapse Free Survival		N(n)	HR	%95 CI	*p*-value
All Breast Cancer All Samples					
Nup98	low	1984(671)	1		
	high	1984(602)	0.95	00.85–1.06	0.37
All Breast Cancer Untreated cases excluded					
Nup98	low	940 (291)	1		
	high	940 (333)	1.35	1.15–1.58	0.00016***
TNBC All Cases					
Nup98	low	128 (37)	1		
	high	128 (47)	1.37	0.89–2.09	0.15
TNBC Untreated cases excluded					
Nup98	low	58 (13)	1		
	high	58 (25)	2.07	1.05–3.97	0.025*

**Table 5:** Statistical analysis of the correlation between NUP98 expression (dichotomised based on NUP98 expression above (high) and below the median (low)) and pathological response in publicly available neo-adjuvant datasets (GSE6861, GSE22093 and GSE20271)

	Relative Risk	Odd’s Ratio	Chi-Squared *p*-value	Fisher’s exact *p*-value
GSE6861 FEC	2.5	4.8	0.0028	0.0057**
GSE6861 TET	1.277	1.481	0.3544	0.4018
GSE22093 FEC/FAC	1.289	2.67	0.0317	0.0463*
GSE20271 FEC/FAC	1.702	3.506	0.0323	0.0483*

## Discussion

In this study we have identified NUP98 as a novel biomarker of response to SoC, DNA-damaging chemotherapy in TNBC. We have shown that both higher gene and protein expression are associated with poor clinical outcome in TNBC. Investigation into the role of NUP98 as a biomarker in breast cancer as a whole showed similar trends to TNBC especially within Luminal B,HER2 negative cases. Using publicly available data, we could show that NUP98 did not predict response in the absence of systemic treatment or in the context of taxane-based chemotherapy but was highly predictive of response to anthracycline based regimens.

The nucleoporin protein, NUP98, is an essential part of the nuclear pore complex (NPC); a very large (> 100 MDa) protein complex involved in the transport of molecules across the nuclear envelope. Nucleoporins are one of four key factors affecting nuclear transport. The role of these as well as Ran, karyopherin and NLS/NES are reviewed elsewhere [18, 19]. NUP98 is a peripheral nucleoporin and found on both the nuclear and cytoplasmic sides of the NPC [10] and is thought to have different functions depending on binding partners, such as NUP88 and NUP96 which are found on the cytoplasmic and nuclear sides of the NPC, respectively [20]. NUP98 has also been shown to interact with RAE1 and play an important role in the export of mRNA from the nucleus [21]. In addition to its role in nuclear transport, NUP98 has been shown to play an important role in gene regulation through its ability to dynamically interact with the genome and regulate chromatin structure and transcriptional memory [22–25]. Furthermore, it is involved in mitosis though regulation of the anaphase promoting complex and microtubule dynamics [26, 27].

The first link between NUP98 and cancer came from its identification as a fusion partner with HOXA9 in AML patients [28, 29]. Since then, multiple other fusion partners (e.g. JARID1A and SETBP1) have been identified in multiple types of haematological malignancies (reviewed in [30, 31]). The known functions of these fusion partners implicate the change in chromatin structure and subsequent regulation of transcription in NUP98-induced leukemogenesis.

NUP98 itself has also been shown to play an important role in regulating the nuclear to cytoplasmic trafficking of GALECTIN3, a oncogene with known roles in cell growth, adhesion, migration, invasion, angiogenesis and apoptosis [32]. More recently, a study has revealed novel role for NUP98 as a potential tumour suppressor in hepatic cancer [33]. Here the authors showed that NUP98 functions during genotoxic stress to protect specific p53-induced targets (such as p21^WAF1^ and 14–3-3σ) from exosome-dependent degradation by binding to the 3’UTR of the p53 target gene. Loss of NUP98 resulted in decreased p21^WAF1^ expression and reduced senescence in response to genotoxic stress which could in turn lead to tumourigenesis though loss of wild-type p53 function. They went on to show that NUP98 mRNA expression is reduced in patient samples and correlated with p21^WAF1^ expression. Our findings, however, indicate an oncogenic role for NUP98 and this may be due to the fact that, in contrast to liver cancer, the vast majority (> 80%) of TNBC cases harbour gain of function p53 mutations [34, 35]. It is possible that, in this scenario, NUP98 functions to stabilise a subset of mutant p53 target genes and thus promote tumour resistance to chemotherapy. This may also underpin the association between NUP98 and poor outcome in patients with LuminalB,HER2- disease as this subgroup is known to harbour high p53 mutation rates relative to LuminalA (41% vs 17% respectively) [36]. Further studies involving RNA-IP would be required to identify potential mutant p53 target genes and assess their role in response to treatment and tumourigenesis. Furthermore, the cytoplasmic pattern of expression observed in our TNBC cases differs from the normal expression pattern of NUP98 where it is confined to the NPC or nucleoplasm. This suggests loss of its normal functions in regulation of chromatin structure and nuclear transport in favour of its potential “mRNA chaperone” function.

The potential interplay between NUP98 and mutant p53 may also explain why NUP98 does not predict response to taxane based regimens. A number of studies have shown that mutant p53 confers resistance to anthracyclines [37, 38], while not changing [39] or increasing sensitivity [40] to taxanes. This was tested in a phase III randomised clinical trial but failed to demonstrate predictive power [41]. However, the yeast assay used to determine p53 status does not distinguish between different types of p53 mutations and therefore a sequencing approach would be required to determine if a specific gain of function effect was present. Furthermore, while the main analysis focussed on breast cancer as a whole, varied responses were seen when analysed in the context of breast cancer subtypes, with TNBC showing a different trend to the other subgroups [15]. This subtype dependent effect has also been observed in other studies [42] and is consistent with the correlation between NUP98 and survival in the different subtypes.

Overall this study highlights the potential role for NUP98 as a biomarker of response to SoC chemotherapy in TNBC. To the best of our knowledge, this is the first time NUP98 (as opposed to a fusion partner) has been associated with poor outcome, suggesting an oncogenic role for the protein in this context. Other members of the nucleoporin family have been linked with cancer including Tpr, NUP62, NUP214 and NUP358/RanBP2 [31] with NUP88 and more recently, NUP43 specifically linked to poor outcome in breast cancer [43, 44]. Analysis of our TNBC cohort shows that while there was no correlation between NUP98 and NUP43 expression, NUP43 is associated with good outcome both in terms of higher expression (*p* = 0.0015) and RFS (HR3.53 (95%CI 1.478–7.99) *p* = 0.0045) (Additional file 3 Figure S2B). This observation warrants further analysis in a future study to delineate the factors underpinning the differing results and the significance of NUP43 as a potential biomarker in breast cancer.

## Conclusion

In conclusion we have identified a novel biomarker that can predict response to anthracycline based chemotherapy in TNBC. The ability to prospectively identify patients who are less likely to respond to SoC chemotherapy is a vital step in improving the overall survival of these patients. Furthermore, given the significant expression of NUP98 in both DCIS and invasive breast cancer relative to normal breast provides further utility for this biomarker in the early diagnosis of the disease. Further understanding of the molecular mechanisms of the role of NUP98 in tumourigenesis and how it modulates response to treatment may provide novel treatment strategies to personalise treatment and improve the outcomes for women with TNBC.

## Additional files

Additional file 1: **Table S1.** Clinical and pathological data of patient samples within the 2nd TNBC cohort. **Table S2.** Contingency table of NUP98 expression (dichotomised based on NUP98 expression above (high) and below the median (low)) and pathological response in publicly available neo-adjuvant datasets (GSE6861, GSE22093 and GSE20271). (PPTX 69 kb)

Additional file 2: **Figure S1.** (A) Kaplan Meier plot of overall survival of TNBC patients from in-house datasets dichotomised based on NUP98 gene expression above (high) and below the median (low). (B) Kaplan Meier plot of overall survival stratified based on absent (0), low (1) or high (2) NUP98 IHC expression in the TNBC TMA with matched gene expression. (C) Kaplan Meier plot of overall survival stratified based on absent (0), low (1) or high (2) NUP98 IHC expression in the 2nd TNBC TMA. (D) Kaplan Meier plot of overall survival stratified based on presence or absence of NUP96. (PPTX 170 kb)

Additional file 3: **Figure S2.** (A) Kaplan Meier plot of overall survival dichotomised based on NUP88 gene expression above (high) and below the median (low) in the in-house TNBC gene expression dataset.(B) Box and whisker plot of NUP43 gene expression in good and poor outcome samples in the in-house TNBC gene expression dataset. (C) Kaplan Meier plot of relapse free survival dichotomised based on NUP43 gene expression above (high) and below the median (low) in the in-house TNBC gene expression dataset. (PPTX 156 kb)

## Abbreviations

ERα: Estrogen receptor alpha
FEC: 5-Fluorouracil, Epirubicin and Cyclophosphamide
FFPE: Formalin fixed paraffin embedded
H&E: Haematoxylin and eosin
HR: Hazard ratio
IHC: Immunohistochemistry
NPC: Nuclear pore complex
OS: Overall survival
pCR: Pathological complete response
PR: Progesterone Receptor
RD: Residual disease
RFS: Relapse free survival
SoC: Standard of care
TMA: Tissue microarray
TNBC: Triple negative breast cancer
