# Supplementary material for: NUP98 – a novel predictor of response to anthracycline-based chemotherapy in triple negative breast cancer
Source: BMC Cancer. 2019 Apr 2;19:236. doi: 10.1186/s12885-019-5407-9 (PMC6444590; doi:10.1186/s12885-019-5407-9)
Supplement: Supplementary file 1 — Table S1. Clinical and pathological data of patient samples within the 2nd TNBC cohort. Table S2. Contingency table of NUP98 expression (dichotomised based on NUP98 expression above (high) and below the median (low)) and pathological response in publicly available neo-adjuvant datasets (GSE6861, GSE22093 and GSE20271). (PPTX 69 kb) [file 12885_2019_5407_MOESM1_ESM.pptx]

## Slide 1
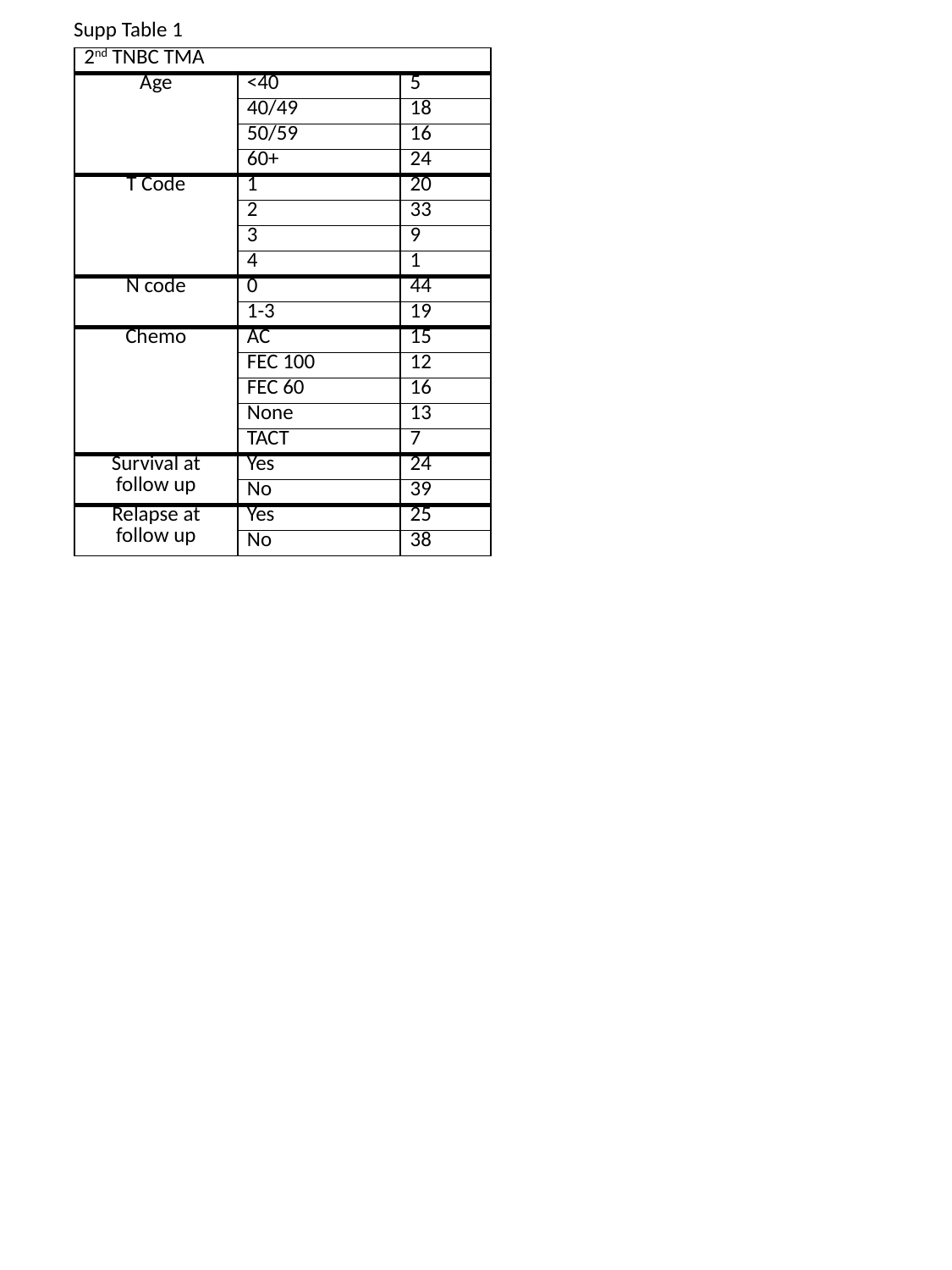

Supp Table 1
| 2nd TNBC TMA | | |
| --- | --- | --- |
| Age | <40 | 5 |
| | 40/49 | 18 |
| | 50/59 | 16 |
| | 60+ | 24 |
| T Code | 1 | 20 |
| | 2 | 33 |
| | 3 | 9 |
| | 4 | 1 |
| N code | 0 | 44 |
| | 1-3 | 19 |
| Chemo | AC | 15 |
| | FEC 100 | 12 |
| | FEC 60 | 16 |
| | None | 13 |
| | TACT | 7 |
| Survival at follow up | Yes | 24 |
| | No | 39 |
| Relapse at follow up | Yes | 25 |
| | No | 38 |

## Slide 2
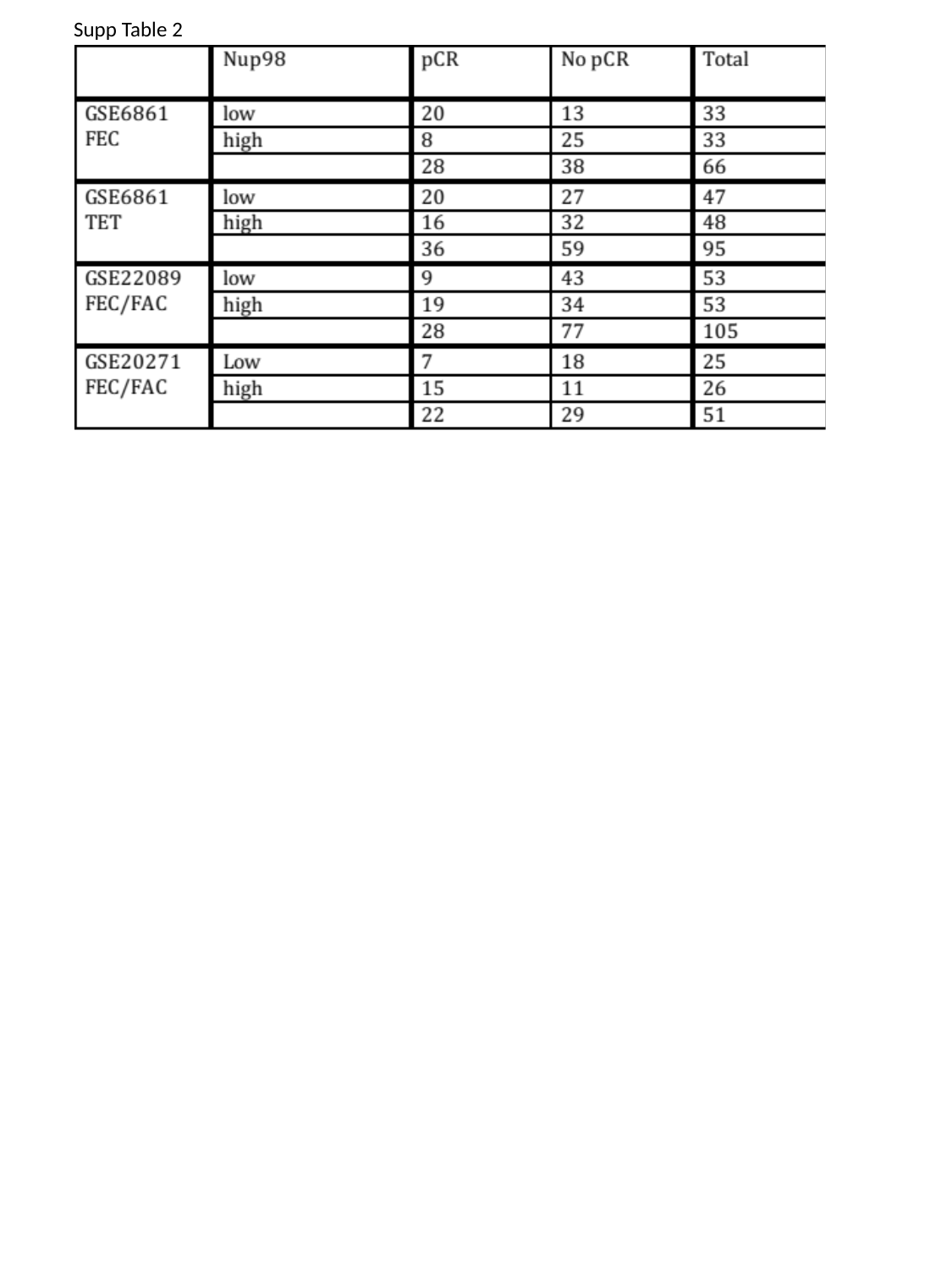

Supp Table 2
