# Supplementary figures and images for: NUP98 – a novel predictor of response to anthracycline-based chemotherapy in triple negative breast cancer
Source: BMC Cancer. 2019 Apr 2;19:236. doi: 10.1186/s12885-019-5407-9 (PMC6444590; doi:10.1186/s12885-019-5407-9)

## Slide 1
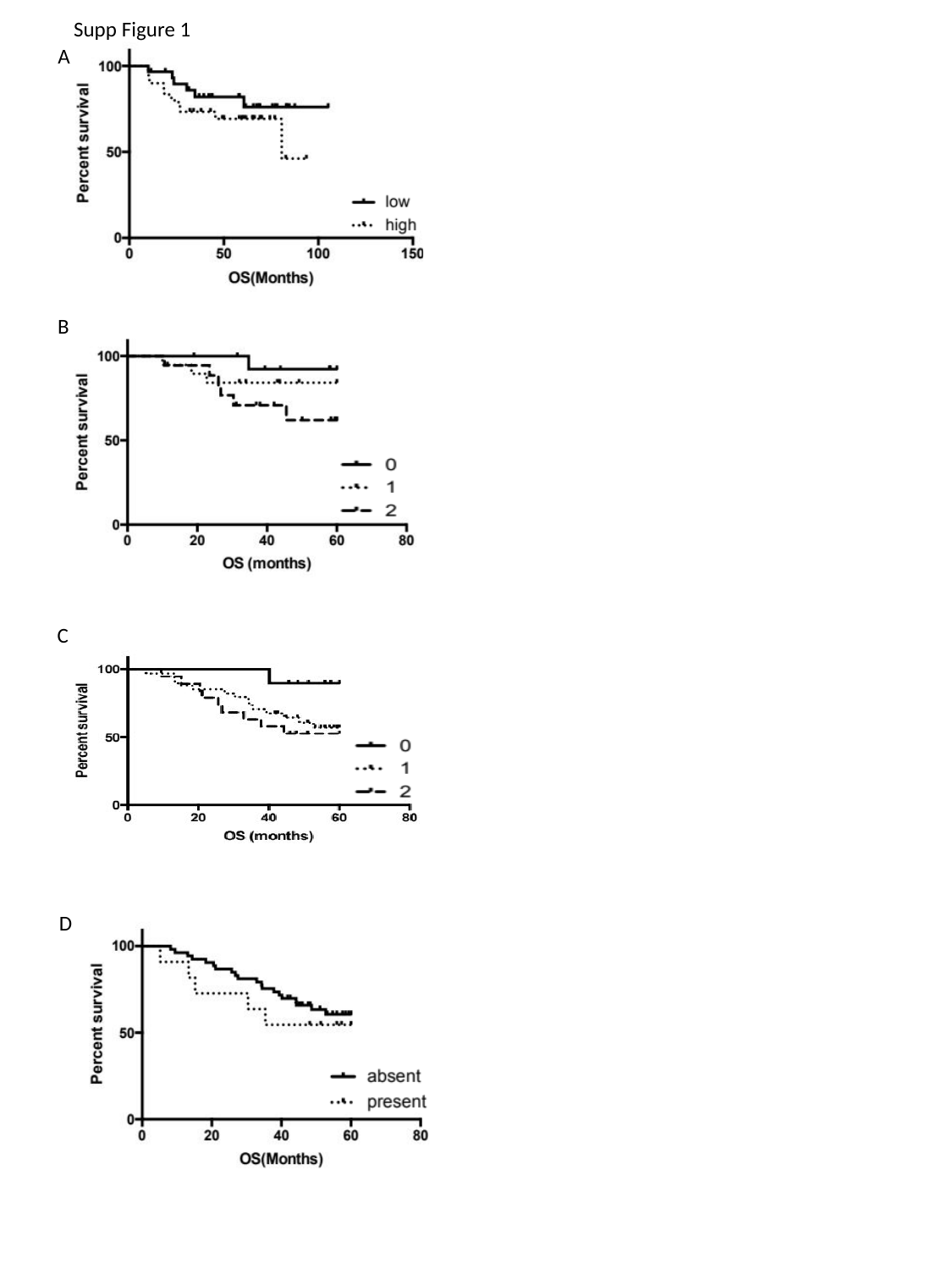

Supp Figure 1
A
B
C
D

Supplement: Supplementary file 2 — Figure S1. (A) Kaplan Meier plot of overall survival of TNBC patients from in-house datasets dichotomised based on NUP98 gene expression above (high) and below the median (low). (B) Kaplan Meier plot of overall survival stratified based on absent (0), low (1) or high (2) NUP98 IHC expression in the TNBC TMA with matched gene expression. (C) Kaplan Meier plot of overall survival stratified based on absent (0), low (1) or high (2) NUP98 IHC expression in the 2nd TNBC TMA. (D) Kaplan Meier plot of overall survival stratified based on presence or absence of NUP96. (PPTX 170 kb) [file 12885_2019_5407_MOESM2_ESM.pptx]

## Slide 1
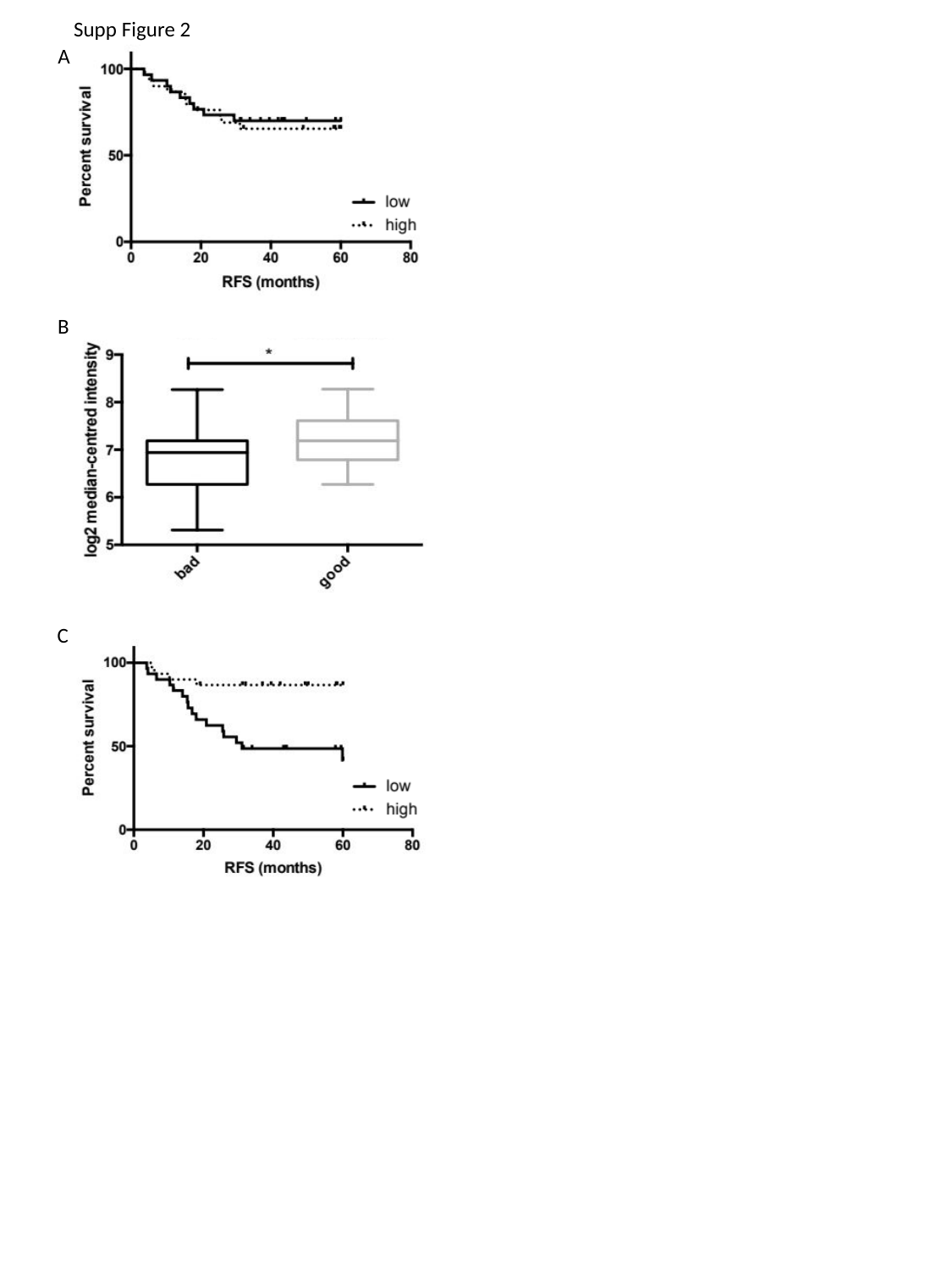

Supp Figure 2
A
B
C

Supplement: Supplementary file 3 — Figure S2. (A) Kaplan Meier plot of overall survival dichotomised based on NUP88 gene expression above (high) and below the median (low) in the in-house TNBC gene expression dataset.(B) Box and whisker plot of NUP43 gene expression in good and poor outcome samples in the in-house TNBC gene expression dataset. (C) Kaplan Meier plot of relapse free survival dichotomised based on NUP43 gene expression above (high) and below the median (low) in the in-house TNBC gene expression dataset. (PPTX 156 kb) [file 12885_2019_5407_MOESM3_ESM.pptx]
